# Corrective Justice as A Principle of Criminal Law: A Prolegomenon

**DOI:** 10.1007/s11572-017-9447-4

**Published:** 2017-10-23

**Authors:** Andrei Poama

**Affiliations:** Faculty of Governance and Global Affairs, Institute of Public Administration, Leiden University, Campus Den Haag, Netherlands

**Keywords:** Corrective justice, Criminal law, Criminalization, Private property, Rape, Inchoate offences, Private/public law divide, Civil/criminal law divide

## Abstract

This article argues that corrective justice is an adequate principle of criminalization. On my interpretation, corrective justice holds that, in order for an action to count as a crime, there needs to be a plausible normative story about an offender having violated the interests of a victim in a way that disturbs their relationship as equal persons and a subsequent story about responding to crime in a way that corrects this disturbance. More specifically, I claim that corrective justice is concerned with the protection of interests that persons have in owning private goods throughout standard interactions with other persons. The argument proceeds in three steps. First, I specify the subject-matter that principles of criminal law need to ground and provide an outline of the idea of corrective justice. Second, I show that corrective justice can account for the main cases of crime and the salient modes of criminal responsibility. I also argue that corrective justice can make sense of two *prima facie* recalcitrant types of cases (rape and inchoate offenses). Third, and finally, I address two objections to my corrective justice theory of criminal law. The first concerns the implications corrective justice has for locating criminal law along the private/public law divide. The second objection raises the putatively problematic consequences corrective justice has for understanding the separation between criminal and civil law.

## Introduction

Any normative theory of criminal law needs to answer two questions: first, “what ought to be criminalized?” and second, “who ought to be held responsible for acting criminally?” The first question concerns the proper limits of criminal law; the second one looks at the contours of criminal responsibility. This article argues that, adequately understood, the principle of corrective justice can provide a unified answer to both of these questions. The basic claim behind the principle of corrective justice is that, in order for an action to count as a crime, there needs to be a plausible normative story about an offender having violated the interests of a victim in a way that disturbs their relationship as equal persons and a subsequent story about responding to crime in a way that corrects this disturbance. I will argue for a specific interpretation of corrective justice, one that requires that crimes consist in interpersonal violations of those interests that matter for construing the relationship between the victim and the offender as one between persons viewed as owners of private goods. More specifically, I will claim that corrective justice is concerned with the protection of interests that persons have in owning their goods throughout standard interactions with other persons. Since some actions unwarrantedly violate private property in various ways, they call for criminalization.^1^

This article is prefatory to an alternative way of thinking about criminal law. Being a prolegomenon, my account is bound to remain relatively rough, and will therefore be unable to address all (or even all of the important) implications of justifying criminal law on a corrective justice basis. For example, I will not provide a full account of what it means to think about relations between persons as relations between proprietors of private goods. Nor will I offer a general justification of private property or examine whether corrective justice can fully capture the condemnatory dimensions of criminal law. These are topics whose exploration requires a longer treatment than I can offer here.

The article proceeds as follows. In Sect. 2, I offer some methodological considerations on justification and criminal law. In Sect. 3, I provide a working conception of corrective justice that highlights the idea of responsibility suitable for understanding corrective wrongs and properly specifies the substance of these wrongs. In Sect. 4, I examine whether, thus understood, corrective justice can account for the main cases of crime. I do this by scrutinizing the offense categories contained in the Model Penal Code (MPC). Finally, in Sect. 5, I address two general objections. The first concerns the implications corrective justice has for locating criminal law along the private/public law divide. The second objection raises the putatively problematic consequences corrective justice has for understanding the separation between criminal and civil law. Both objections are defeasible—or so, at least, I will argue.

## Justifying Criminal Law

Any justificatory theory of criminal law raises important questions about the choices involved in specifying both its *justificandum* and its *justificans*. On the side of the *justificandum*, we will have to choose between the set composed of all the existing criminal offenses and an adequately specified sub-set belonging to this wider set. On the side of the *justificans*, we will have to be able to come up with a plausible story about why any given principle represents an attractive standpoint for justifying criminal law.

Taking all the existing criminal offenses as the *justificandum* is problematic for at least two reasons. First, it supposes that we have a clear picture about the totality of actions, conducts, and states of affairs that are criminalized at a particular moment. Such a picture is missing. As Douglas Husak points out in relation to US federal criminal law, “offenses are hard to find or enumerate” and, given the proliferation of regulatory provisions and their capacity to trigger criminal sanctions, “the situation gets worse each month.”^2^ The inability to count—much less to describe—the things that are currently criminalized is not specific to US federal law, nor is it, for that matter, specific to US criminal law.^3^

The second problem is that specifying the *justificandum* in terms of the totality of legally enforceable offenses rests on an insufficiently moralized view of criminal law. This is because it supposes that criminalization principles should be able account for all criminal offenses, even when they result from purely practical requirements (which is arguably the case with “proxy crimes”) or are the products of ideological fads (which was quite clearly the case during with the legislation that was instrumental to the “war on drugs” policies). By opening the *justificandum* to irrelevant or dubious considerations, this all-encompassing approach potentially invites us to justify the unjustifiable.

But, if the relevant *justificandum* is less than the totality of criminal law, we need a method for specifying it. The method I privilege is to look at the results of systematic attempts to codify the criminal law.^4^ Codes represent relevant *justificanda* for at least six reasons. First, they are based on considerations defended on an explicit normative basis, not advanced in virtue of legal inertia. Second, codes typically contain provisions that most criminal justice practitioners consider to be legitimate from a standpoint internal to criminal law itself. Thus construed, they are *meant* as an answer to the question of criminalization and are actually used by practitioners when deciding what the substantive criminal law is on a particular matter. Third, codes usually point to substantial areas of agreement on things deemed criminalizable. To put it in Markus Dubber’s terms, “if there is such a thing as a common denominator in contemporary American criminal law, it is the Model Penal Code.”^5^ Fourth, given their limited length, codes specify the substance of criminal law based on principles of normative priority,’ with the most important offense categories included in the code and the rest left to subsequent legislation. Fifth, given their systematic nature, codes are comparatively clearer and more coherent than common law, thus guaranteeing that the chosen criminal law *justificandum* will satisfy a minimal rationality test.^6^ Sixth, and equally importantly, codes are the result of a process of deliberation that includes several members of the social and professional community, a factor that plays in favor of their practical relevance.

Though it is relatively easy to specify the relevant criminal law *justificandum*, choosing an appropriate *justificans* is less obvious. This is because different theorists’ considered judgments about crime are often at odds with each other. Sometimes this is the result of the professionalization of philosophy. Philosophers and theorists of criminal law are expected to think in increasingly innovative and challenging ways about their subject-matter, and are thus sometimes brought to overplay the differences between their views. But sometimes disagreement is bedrock, with the constant opposition between the retributive and the utilitarian modes of thinking about justice being a symptom of this difference.

I will not attempt to counterfactually engineer readers’ intuitions about the rationale of criminal law. Bedrock intuitions are difficult to change, even in cases where one becomes rationally convinced of their problematic implications at the moral or political level.^7^ Rather, my objective is to show that, even when my intuitions about the grounds for criminalizing an action or behavior are not entirely shared, corrective justice will be able to provide a reasonable candidate for understanding criminal law.

The focus on corrective justice is premised on the idea that, no matter what other wrongs crimes instantiate, their distinctive (and intuitively available) wrong consists in the violation committed by an offender in relation to his victim. A crime is something an offender does to a victim, in a way that disturbs their equal relation and asks for a correction of the ensuing inequality. This thought will be further developed in the following section.

For the purposes of this section, suffice it to say that my emphasis on corrective justice is an attempt to articulate a normative vocabulary that will take seriously the offenders’ and the victims’ (defensible) views on criminal law. Articulating the *justificans* of criminal law around the relation between victims and offenders intersects with some past philosophical projects—for instance, with Randy Barnett’s restitutionary theory of criminal justice at the end of the 1970s or Charles Abel and Frank Marsh’s restitution-based account of punishment during the mid-1980s.^8^ It also shares Niels Christie’s contention that criminal law should be designed so that it does not “steal the conflict” between victims and offenders.^9^

More generally, my focus on corrective justice is meant as a device for giving normative credence to a certain kind of complaint advanced by the representatives of victims and offenders during the last three to four decades in the Anglo-American world and elsewhere. On the side of the victims, the complaint is that criminal law has unwarrantedly substituted the state’s interest for the victim’s interests. On the side of the offender, the complaint is that criminal law has been extended to include actions—for instance, prostitution or euthanasia—or state of affairs—like drug possession—that should not be criminalized. When read jointly, these two complaints ground the view that crime should be primarily about the victim’s interest and that, when there is no victim, there is no stringent reason for criminalization. Corrective justice tries to ground these two views simultaneously.

Focusing on corrective justice does not mean that other principles might not be at play in justifying criminal law. I do not argue that corrective justice is the only plausible candidate for thinking about criminalization. In this, I share R.A. Duff’s diagnosis that there is something reductionist about any “grand, unitary theory of criminal liability,” and I believe that, given the complexities of criminal law and the plurality of normative outlooks that come with it, “such theorizing is doomed to ultimate frustration.”^10^

Notwithstanding normative pluralism, I will argue that the principle of corrective justice should be made salient in the justification of criminal law. This is because corrective justice offers an adequate principle for most offenses that form the core of criminal law. There are areas of criminal law—in particular, those covering regulatory offenses—corrective justice will probably not be able to ground without being somewhat stretched, both conceptually and normatively.^11^ Whether such stretching will be needed or not, the argument I will defend concerns only the core (and not the periphery) of criminal law, as that core is reflected through my code-based specification of the criminal law *justificandum*.^12^ But, before arguing that corrective justice can account for the core of crime, we need to clarify what corrective justice is. This is the task I turn to in the following section.

## Corrective Justice: A Working Conception

No matter how one construes corrective justice, there is virtual consensus that its underlying intuition is that a person who causes wrongful harm to another should correct it.^13^ In order for this intuition to be properly grounded, one needs to show that the relevant notion of wrongful harm is in some sense correctable, i.e., that it can fall under a description that makes sense of an action (or set of actions) as a mechanism for correcting that wrongful harm. The same moral intuition requires that we can offer reasons that that harm is wrongful. Furthermore, it demands that we understand why correcting certain harms is an obligation most adequately incurred by its author and not by another agent who might be somehow related to him—say, by the author’s parents or by the state that holds him under its authority. Any convincing conception of corrective justice is an attempt to make sense of this intuition by considering how its implications hang together within the same normative framework.

More specifically, any theory of corrective justice ought to be able to specify three things: (1) the content of the relations that fall within the scope of corrective justice, (2) the nature of the relata that give rise to those relations, and (3) the manner (or mode) in which the relata bring about these relations. Specifying (1) and (2) will delimit the scope of the harms with which corrective justice deals. Specifying (2) and (3) will tell us why harms are wrongful and point to the notion of responsibility relevant to corrective justice.

There is more than one strategy for jointly meeting these three specificatory requirements. For example, Ernest Weinrib argues that we should think about (1) in terms of unjust relations of formal inequality between persons, about (2) in terms of persons as holders of abstract rights, and about (3) in terms of the responsibility one person (the author) incurs for having violated another person’s (the victim) abstract rights.^14^ Jules Coleman adopts a different strategy, whereby (1) is specified as the harmful losses some agents impose on others, (2) as agents having legitimate interests, and (3) as the causal responsibility agents have for correcting the losses they have imposed on others, typically through compensation.^15^

I will not criticize these two specificatory strategies here. For the purpose of this article, suffice it to say that, in both cases, thinking about (2) in terms of holders of abstract rights or legitimate interests is too encompassing, because it renders corrective justice roughly co-extensive with political morality.^16^ Another critique is that Coleman’s interpretation results in a mismatch between the proposal that (2) should be widely construed as holders of legitimate interests and the parallel requirement that (1) and (3) should nonetheless be limited to the compensation of harmful losses.^17^

The point of this second critique is that, in order for the elements of a conception of corrective justice to hang together coherently, the proper specifications of (1), (2), and (3) should be kept at the same level of generality. We cannot interpret (2) in terms of holders of legitimate interests and insist that (1) should be limited to a sub-set of the actual setbacks imposed on those interests, i.e., to harmful losses. Doing so means that we have either underspecified (2) or overspecified (1), and ultimately renders one’s interpretation of corrective justice conceptually bumpy.

In the rest of this section, I will argue for an interpretation of corrective justice that points to an internal connection running through (1), (2), and (3) and places them at the same level of generality. For lack of space, I will offer only a working conception of corrective justice, i.e., one that does not flesh out all the relevant dimensions of my interpretation of (1), (2), and (3), but offers at least some suggestions as to how the specification of these three elements could be carried further.

As stated at the beginning of this article, I understand corrective justice as a principle that deals with actual inequalities that one person (the author) directly imposes on another (the victim), with both persons construed as rightful holders of certain private goods. When such inequalities are unjustly imposed, the author incurs an obligation to correct that inequality. This means that (1) should be interpreted in terms of actual violations of interests specific persons have in controlling certain private goods, (2) should be understood in terms of persons as rightful owners of those goods—or, in short, as holders of private property, and (3) should be construed as the responsibility an author has for having brought about an outcome consisting in the violation of the victim’s interest in controlling certain goods.

This interpretation of corrective justice might sound stipulative. It is not. The interpretation is not meant as an analytic construct, but rather as a diagnosis for a cluster of actions tied by similar traits and as a solution for righting the wrongs generated by these actions. The diagnosis and the solution were first sketched by Aristotle. In what follows, I will provide a brief rational reconstruction for Aristotle’s discussion of corrective justice, and chart some reasons why my reconstructive interpretation is attractive for us today.

In Book V of the *Nicomachean Ethics* and Book IV of the *Eudemian Ethics*, Aristotle presents corrective justice as a distinctive principle that “plays a rectifying part in interactions” between persons.^18^ Corrective justice regulates two kinds of such interactions. The first kind is voluntary, i.e., it has its origin in a mutually voluntary interaction, and includes dealings like “sale, purchase, usury, pledging, lending, depositing, letting.”^19^ The second is involuntary, i.e., it does not depend on an initial voluntary dealing, and it further subdivides into clandestine and violent interactions. Among the clandestine, Aristotle includes “theft, adultery, poisoning, procuring, enticement of slaves, assassination, false witness;” for the violent, he enumerates “assault, imprisonment, murder, robbery with violence, mutilation, abuse, insult.”^20^

There is a cluster of traits that these interactions share. First, they are all the doings of an author and, as such, constitute the subject of moral evaluation. Of one of the two persons involved in any specific interaction, one’s mental states (the author’s) play a central role in initiating it. In this sense, all these interactions are voluntary from their author’s standpoint. This is to say that the author’s mental states—whether, as Aristotle indicates later in Book V, they refer to the author’s deliberate intent, carelessness, or negligence^21^—are needed to explain why the interaction took place. For instance, not fulfilling a sales contract is best explained in terms of (at least) one of the parties deliberately or carelessly refraining from satisfying one’s contractual obligations. Similarly, a person being insulted can be best explained in terms of another person’s deliberately, recklessly, or negligently harmful speech act.

The point about voluntariness is that the interactions falling under the corrective justice rubric are not aptly described as happenings or general social facts, but as actions performed by specific persons. Unlike happenings or social facts, interactions need authors. Because it applies only to interactions, corrective justice does not deal with harmful events *tout court*. Corrective justice does not cover harms resulting from natural events like storms or earthquakes. Nor does it, for example, concern the harm that can be incurred by a person whose life expectancy is lowered by the surrounding social conditions. This is because neither of these two kinds of events can be plausibly explained in terms of a single person’s mental states.

The second trait specifying these actions is that they all result in a specific outcome that can be aptly described as harmful for a person other than the author of the harm. An insult is an outcome suffered by the insulted, not the insultee. This is to say that, absent a harmful outcome incurred by a victim, an author’s action, as deliberated or as careless it might otherwise be, does not fall under the scope of corrective justice. Correctively relevant interactions need more than authors. They also need actual victims who are harmed as a direct result of those interactions.

The importance of outcomes for corrective justice has been interpreted in terms of outcome-responsibility. The expression was introduced and examined by Tony Honoré and more recently developed by Stephen Perry.^22^ The main claim behind the idea of outcome-responsibility is that the agents whose relevant mental states play a central role in accounting for the outcome of an interaction incur a special responsibility to correct for the harm of those outcomes.^23^

Outcome-responsibility supposes that there is a plausible story to be told about the way in which the author’s mental state—say, intent or recklessness or negligence—is connected to and accounts for the result of an interaction. Outcome-responsibility is premised on the contention that an author’s mental state commits him to answer for his action’s outcomes. To put it in Perry’s terms, “the basic idea is that, as a moral agent, I must take responsibility for certain of the outcomes of my actions, simply because they are the outcomes of *my* actions.”^24^ The possessive pronoun refers to the agent as a holder of particular mental states—intent, recklessness, negligence, and so on—that are outcome-relevant.

Perry further argues that we should think about outcome-responsibility in terms of avoidability. For the purposes of my working conception of corrective justice, I will follow Perry’s proposal in this respect. Avoidability posits that an agent is responsible for a particular outcome *because* she had the capacity to foresee it and *because* she did not take the steps that she could have taken to avoid it. Foreseeability and avoidability are, on this conception, individually necessary and jointly sufficient conditions for a person to be outcome-responsible.

Specifying corrective justice in terms of outcome-responsibility does not imply that intent or culpability become irrelevant. What it means is that the mental states in virtue of which a person can be considered outcome-responsible should not be limited to intent, at least not as long as there are other mental states in terms of which foreseeability and avoidability can be cashed out. In particular, it means that mental states like recklessness and negligence are plausible ways of understanding the responsibility that is relevant for corrective justice. Though different from choice, recklessness and negligence are apt specifications of mental states whereby an agent can be considered to have brought about a harm both foreseeably and avoidably. In the case of recklessness, the harm is foreseen and avoidable. In the case of negligence, the harm is foreseeable and avoidable.^25^

From a corrective justice standpoint, the difference between choice, recklessness, and negligence is not a difference between responsibility (for choice) and its absence (in the case of recklessness and negligence). Rather, it is a difference between different kinds or modes of outcome-responsibility. In the case of choice, the author can be said to be responsible because he did not only foresee and avoid harm, but deliberately acted *to* cause it. By doing so, the author exerted a kind of control in causing the outcome that is absent from the instances where he acted recklessly or negligently.

A plausible mechanism accounting for these different modes of outcome-responsibility is the author’s disposition to cause harm. In the case of choice, the author is entirely committed to causing harm, and invites us to interpret his action in terms of an underlying disposition to harm his victim and thus act in a correctively unjust manner. When the author chooses to harm his victim, he does not simply harm her, but harms her *because* she is disposed to do so. In the case of recklessness or negligence, his actions can be interpreted as displaying at least an insufficiently developed disposition to act in ways that are not harmful for others.

Corrective justice is thus sensitive to the characterological dimension of responsibility. A person should be held responsible because her actions are in some way reflective of a relevant component of her character or, in cases where the act might seem out of character, to allow herself to act in certain ways.^26^ The reason that mental states matter is that they inform us about the underlying disposition that person has. A person does not simply happen to negligently injure another while driving. The same is the case when she injures another recklessly or deliberately.

When a person acts intentionally, recklessly, or negligently, she tells us something about her moral character. Corrective justice is only concerned with outcomes that harm victims, but, *once those outcomes occur*, it is interested in them as results of the author’s dispositionally sustained mental states. In order for a person to be outcome-responsible for a harm, she “must be in” for something, and that something is adequately explained in terms of her mental states reflecting a moral disposition to act in a specific way. Put differently, corrective justice is a virtue—that is, a trait of character—a person will possess to various degrees. We can say that a person is correctively vicious when she deliberately intends to bring about a corrective harm and that she is not sufficiently correctively virtuous when she causes that harm recklessly or negligently.

These first two traits restrict the scope of corrective justice to interactions that concern two persons (the author and the victim) and result in harms for which the author can be held outcome-responsible in a dispositionally relevant way. But specifying the content of the victim-offender relations falling under corrective justice and the outcome-responsible way in which these relations are caused is insufficient. The notion of harm is still left underspecified. The first two traits do not tell us enough about the kinds of harms that matter correctively. Most importantly, they do not tell us why these harms are wrong. Take the example of a (consented) surgical intervention. The patient can be said to be harmed by the doctor’s scalpel and the doctor can be considered outcome-responsible for the scalpel’s cut. But this is hardly a paradigmatic instance of a corrective wrong.

We therefore need another trait that explains why the cases we tend to intuitively include under the rubric of corrective justice are *wrongful* harms. This wrong-making trait, I suggest, resides in the fact that corrective harms typically violate the interest the victim has in the property of a certain number of personal goods. Corrective wrongs target victims *qua* owners of their personal goods. One paradigmatic instance of a corrective wrong is theft. When the offender steals, she wrongly undermines the victim’s interest in the property of her stolen good. What makes theft wrong is the thief acting in a way that disregards the victim being the exclusive owner of that particular good. Stealing a good is both harmful, insofar as it undermines the welfare (potentially) generated by the property of that good, and wrongful, insofar as it brings about the harm through an action that does not address the victim *qua* owner of the good.

Harmful outcomes are wrong because they disturb the relationship between two persons construed as owners of their private goods. It is clear how that happens in the case of theft, when we are dealing with goods that are physically external to the victim. But corrective justice takes this view about victims and offenders as persons equally entitled to private goods further, and applies it to other instances, such as battery, rape, murder or slander. By doing so, corrective justice invites us to see a person’s body, sex, life, or name as goods over which he has special property claims. Corrective justice is the normative regime whereby persons relate to one other as proprietors having equal claims to their private goods.

To put it in Aristotle’s terms, “when one person has been struck and another struck the blow, or one person has been killed and another did the killing, doing and suffering have been unequally divided, and the punishment is an attempt to restore the equality by depriving the wrongdoer of his gain.”^27^ Aristotle recognizes that *gain* and *loss* might not be terms “appropriate to certain cases—e.g., to the person who inflicts a wound—and loss to the sufferer.”^28^ But this is a terminological, not a normative problem, and it leaves unaffected the fact that, no matter how else we choose to assess the violation of a person’s interest in her life or body, they are *also* wrongful because they violate that person’s property claims.

One might contend that seeing one’s life, body, and sex as goods promotes an outlandish view. This is a point I will come back to. For now, suffice it to say that the view gains credence if we think about it in the context of some our pervasive social and institutional practices. For example, the idea that we are owners of our lives provides a potent explanation for why we do not consider suicide to be an injustice anymore or, at least, we do not construe it as a wrong that gives rise to a justice claim.^29^ Similarly, a person’s property of her body provides us with a useful criterion for distinguishing between the wrong of a person being mutilated and a person voluntarily getting a tattoo or actively contracting for an intervention that somehow affects her bodily integrity.

That being said, construing one’s physical belongings, body, sex, life, or name as private goods requires a closer analysis than I can provide here. I will therefore rely on readers’ intuitive endorsement of this view. I will also refrain from examining the principles that could further ground our private property claims to certain goods. Nor will I examine the considerations that come into play in the regulation of the property arrangements of these goods. This is a task I hope to undertake elsewhere.

My aim in this section was to provide a rough outline for a working conception of corrective justice. This conception argues that corrective justice should be interpreted as the normative regime that deals with the interpersonal violations of people’s property interests in their private goods.

## Corrective Justice and the Model Penal Code

The aim of this section is to examine whether my working conception of corrective justice accounts for the relevant criminal law *justificandum*. For reasons indicated in Section I, I will concentrate on how the corrective *justificans* fares when applied to the MPC. I will do this in three steps. First, I will show that corrective justice can account for most offense categories contained in the MPC. Second, I will contend that what seems like a hard case for corrective justice—namely, rape—is justifiable as a property offense. Third, I will argue that corrective justice offers a suitable basis for the justification of inchoate offenses.

### Corrective Justice, Criminal Liability, and Criminal Offenses

Before moving to the analysis of the offense categories contained in the MPC, it is worth noting that the kind of responsibility relevant for corrective justice—i.e., outcome-responsibility—offers a unified account for the four main kinds of culpability formalized by Section 2.02 of the MPC, namely purpose, knowledge, recklessness, and negligence. This constitutes an explanatory advantage corrective justice has over conceptions that construe choice or intent as the “essence of criminal liability.”^30^ Unlike these accounts, corrective justice does not take the offender’s choice as the privileged mode of criminal liability. Instead, it argues that all it takes for establishing criminal liability is outcome-responsibility. This broader understanding of responsibility makes other mental states—like recklessness and negligence—equally relevant for understanding criminal liability.

With recklessness being the default form of culpability under the MPC—but also under other similar code projects, such as the UK Draft Criminal Code—taking the normative emphasis off choice should constitute an explanatory advantage that corrective justice has over alternative accounts of criminal liability.^31^

Beyond fit, this de-emphasizing of choice as the essence of criminal liability has at least another desirable implication. When we take choice as the essence of criminal liability, we tend to think of offenders acting by choice as “more criminal” than others. For example, Victor Tadros does this when he describes intentional offenders as “*truly* criminal.”^32^ But there is nothing more truly criminal about a person who offends intentionally as compared to a person who offends recklessly or negligently. Intuitively, a petty thief who acts by choice is not more truly criminal than a reckless arsonist. Taking choice as the essence of criminal liability cannot make full sense of this intuition. Construing criminal liability as outcome-responsibility will not only make sense of the above intuition; it will also allow for more fine-grained distinctions between different offenses and, within each offense category, between different degrees of liability.

Establishing criminal liability on an outcome-responsibility basis would take the form of a two-stage test. First, one would need to prove the presence of a wrongful harm violating a person’s interest in a private good. Second, one would need to show that that wrongful harm was foreseeable and avoidable. Following Perry, harm is foreseeable if the agent has the cognitive capacity to foresee it. It is avoidable if she has the practical capacity to avoid it. As indicated in the previous section, the foreseeability and avoidability requirements are satisfied whenever the agent acts with intent (or, in the language of the MPC, on purpose or with knowledge), recklessly or negligently. The first stage of this outcome-responsibility test shows *that* the offender is responsible for the wrongful harm incurred by a victim. The second stage shows *how* she is responsible and selects for the type of appropriate correction—for instance, (material or symbolic) reparation, compensation for the victim’s involuntarily incurred handicaps and, in the case of murder, monetary compensation and normatively appropriate reparative sanctions owed to the victim’s family.^33^

Though they remain quite rough, these considerations suggest that outcome-responsibility offers a plausible way for thinking about criminal liability. In the rest of this sub-section, I will further show that thinking about crime in terms of violations of property interests accounts for most of the substantive offense categories contained in the MPC. The MPC lists 119 offense tokens.^34^ If my interpretation of corrective wrongs as violations of interests in private goods makes sense and we accept that, in relation to us, our bodies, sex, lives, and names should be construed as goods, corrective justice offers an attractive basis for rethinking the substance of criminal law.

Almost half of the 119 offense tokens listed by the MPC—that is, roughly 45%—fall under the description of corrective wrongs outlined in the previous section. These include almost all “offenses against property,” like theft, robbery, burglary, and most offenses “involving danger to the person,” like homicide, assault, reckless endangering, threats, kidnapping, and so on. To the offenses included in these two broad categories, we can add a number of offenses that are legally defined as public offenses, but can be normatively construed as violations of a person’s property interests in the protection of her private goods. These are offenses like disorderly conduct or false public alarms, both of which can be considered harmful to a person’s property interest in her body or, more specifically, mental and physical health. Thinking about these nominally public offenses in terms of violations of private goods increases the percentage of correctively justifiable crimes to 59%.

We can move next to offenses against public administration, a category that amounts to approximately 29% of the substance of the MPC (i.e., 35 offense tokens in all). At first blush, an important number of these offenses—for instance, perjury, tampering with the witnesses, or obstruction of the law—cannot be adequately construed as violations of a person’s interest in personal goods.^35^ But this does not mean that these offenses should be considered unjustified from a corrective justice standpoint. Though corrective justice might not be able to directly ground the criminalization of these actions, it offers indirect reasons for doing so. This is because these offenses might be needed for guaranteeing the stability and efficiency of a system of public administration without which the protection of our interests in private goods could find itself seriously undermined. Put differently, these offenses against public administration could be justified instrumentally, i.e., as means of guaranteeing that the actual violations of our interests in personal goods are sanctioned effectively. This instrumentally valuable level of criminalization increases the percentage of correctively justifiable crimes to 88% of the offenses included in the MPC.

This leaves us with a picture where corrective justice is clearly unable to account for approximately 11.8% of the offenses falling under the provisions of the MPC. This percentage includes some offenses against the family (like bigamy or polygamy), and most of the offenses related to public decency, like obscenity, prostitution, loitering, or prowling. Given that the criminalization of the former remains contentious and that, as it has been argued,^36^ public decency is hardly a principled consideration for any respectable theory of criminal law, the inability of corrective justice to account for this sub-set of offenses looks more like a success than a failure.

### Corrective Justice and the Wrong of Rape

One might object that I have simply taken for granted that almost all offenses that are *not* nominally defined as public by the MPC can be interpreted as violations of a person’s private property. The worry is that thinking about crime in terms of property relations might be particularly objectionable when it comes to violent offenses or crimes against persons. The purpose of this sub-section is to defuse three typical objections that might be raised in connection with a property-centered view of criminal law. I will do this by considering the case of rape. The underlying assumption is that, if these objections can be met in relation to rape, they can *a fortiori* be met in relation to other offenses as well.^37^

On my account, corrective justice requires that we think about rape in terms of a property offense and, by way of implication, to construe sex (or, more specifically, sexual use of one’s body or body parts) as a piece of property. This is not a new argument, and has been most explicitly defended by Donald Dripps.^38^ Arguing that sexual use of one’s body is private property, Dripps proposed that we should remodel the offense of rape by breaking it into two separate offenses, namely sexual assault (in cases where one’s sexual cooperation is illegitimately secured through violence) and sexual expropriation (in cases where the victim refuses, though she is not violently coerced into sexual intercourse). Dripps contends that there are two types of wrong at work in rape. The first one is the violation of the “the interest in freedom from injury.”^39^ The second consists in the violation of “the interest in exclusive control of one’s body for sexual purposes.”^40^ On this account, sexual assault should be criminalized because it contains both violations, while sexual expropriation contains only the latter.

Corrective justice is consonant with Dripps’ commodity theory of sexual use. It holds that certain acts traditionally called *rape* should be criminalized because they violate our property interests in physical integrity, our interest in the private control of our sexual activity, or both. But, unlike Dripps, I do not think that this commits us to construing rape as theft. Metaphor aside, a rapist does not steal his victim’s body in the same way the thief steals his victim’s wallet. The fact that we are physically and psychologically tied to our bodies in ways we are not tied to our wallets renders the theft analogy misdirected. The wrong of rape does not consist in taking away the victim’s body or sexual use of her body. The rapist commits a wrong against his victim’s body (and, typically, the sexual parts of her body) while both her body and sex remain in the victim’s physical possession. Not so with theft.

Though I cannot develop it here, a more apt, if entirely unexplored, analogy for construing rape as a property offense would be to connect it to vandalism, not theft. Like vandalism, rape amounts to a crime that damages, defaces, trespasses, or otherwise *alters* one’s property—one’s home or physical belongings in the case of vandalism, one’s body in the case of rape. Construing rape as vandalism of a person’s body escapes the descriptively odd implications of the theft analogy.

Whether we think about rape as theft or vandalism, thinking about it as a property offense is typically resisted on the basis of three objections. In what follows, I will try to quickly defuse these objections. Note that doing so does not amount to a full defense of the view that rape is a property offense. However, rejecting them does, more modestly, clear the ground for such a view. The first objection is metaphysical. It contends that bodies are constitutive of ourselves as persons and that, consequently, we cannot have a relationship *to* our bodies in the way required by property relationships. Following John Gardner’s formulation, one cannot analogize “what happens to oneself to what happens to what one owns, because oneself cannot be an addition to what one owns.”^41^ This objection confuses the practical significance of bodies to personal identity with their metaphysical necessity for defining it. Moreover, it relies on a highly contentious view of personal identity as physical or physiological identity. Even if one chooses to ignore its more serious impasses, one should accept that, given the sheer biological reality of body renewal, such a view would absurdly imply that, during the same life-time, we are not one but several persons.^42^ The metaphysical objection can therefore be said to be on metaphysically shaky ground.

The second objection is phenomenological. It argues that seeing sex as property cannot capture the victim’s first-person experience of rape. The point of this objection is sound, but misdirected. Though the experience of rape is significant to understanding its wrong, the objection is misdirected for at least three reasons. First, the experience of rape cannot by itself *constitute* the wrong of rape. The moral significance of the experience is not self-validating but premised on the recognition of an independent wrong to which the victim reacts.^43^ Thus, the experience of rape should at best play an evidentiary, not a constitutive, role in assessing the wrong of rape. If this is the case, experiencing rape will not play a semantic role in defining the corresponding crime.

Second, the objection fails whenever experiential accounts of rape report feelings of dispossession. For example, J.M. Bernstein says that victims talk about their rapes in terms of experiences whereby rapists unwarrantedly appropriate *their* bodies.^44^ Though one would have to assess whether such pronoun talk is typical or topical, thinking about rape as a property offense provides a descriptively apt account for at least certain dimensions of victims’ afflictive experiences.

The third drawback of the phenomenological objection is that predicating the wrong of rape on its afflictive experience means that we leave out all the cases of unconscious rape. Insisting on the centrality of experience for defining rape would imply that, if the victim has no experience of rape—for instance, if she is intoxicated or otherwise insentient—there are no strong reasons for criminalizing the corresponding acts. This implication should, on its own, be sufficient for blocking the phenomenological objection.

The third, and final, objection is axiological. It claims that construing bodies and sex as goods involves an unwarranted devaluation of both. As Dripps argues, this objection misses the fact that the property view—and, by extension, my corrective justice view—does not claim that we should treat bodies as consumer goods. Rather, such views argue that bodies and sexual cooperation should enjoy the protections provided for *legal* commodities. To put it in Dripps’ colorful terms, though law treats “poems and pets” as private goods, it does not allow us to treat them like “coffee futures.”^45^ Legal commodification does not as such involve good devaluation. Sometimes, as with poems and pets, it involves exactly the contrary.^46^ More generally, the objection ignores the fact that private property claims are never absolute. The fact that a certain good is private property does not imply that we can handle it exactly as we please. There are normatively sound limits to the use of *any* good, public or private.

My purpose in this sub-section was to weaken some of the worries one might have about understanding crimes as violations of our private property interests. If there are no sound objections to envisaging rape (or its legal equivalent) as a property offense, then, given the higher seriousness of rape in relation to other offenses, there are good reasons to hope that other (less horrific) actions that we intuitively consider criminalizable can also be interpreted as property offenses. The extent to which this view of crime will pass muster depends on how far we are ready to construe other entities—say, one’s name in the case of slander or one’s mental wellbeing and discretionary time in the case of non-sexual harassment—as private goods.^47^

### Corrective Justice: The Case of Inchoate Offenses

Before considering two more general objections, I want to briefly avert the criticism that corrective justice cannot justify the criminalization of inchoate offenses. The thought runs as follows: given that corrective justice is only concerned with actual harms incurred by the victims and that inchoate offenses fail to produce the harms intended by the offender, corrective justice cannot account for inchoate offenses. To put the matter technically, the concern is that a corrective criminal law will be objectivist in an excessively strong sense, with strong objectivism denoting the position whereby actual harms are definitionally necessary for criminalization. There are two considerations that stand against this criticism. First, one can highlight that the MPC takes a generally objectivist standpoint on the substance of criminal law. Most crimes are defined in ways that make results typical components of their *definiens*. For example, theft is defined as the unlawful taking, controlling, or transferring of a piece of property, trespass as entering or remaining in an occupied building or built structure, murder as the causing of another’s death, kidnapping as unlawful restraint of a person, rape as requiring sexual intercourse. Building actual harmful results into the *definiens* of most crimes is not specific to the MPC, and can be found in similar attempts to codify criminal law, such as the UK Draft Criminal Code. Given its emphasis on outcome-responsibility, the principle of corrective justice is particularly well placed to account for all offenses that have a results requirement. This should be an advantage for any theory whose success is measured by its capacity to account for the main cases forming its subject-matter, not for the cases falling in the penumbra.

The second reason is that corrective justice can be plausibly extended to the traditional cases of inchoate offenses, namely solicitation, conspiracy, and attempt. The fact that these crimes do not depend on the actual attainment of their *intended* results does not make them offenses without any results at all. Though the exact substance of the *actus reus* standard remains a matter for ongoing debate, something needs to be done in order for an action to be criminalized as an inchoate offense. As Duff underlines, the inclusion of attempts in the special part of criminal law is indicative of the fact that inchoate liability is paradigmatically predicated “on the objective character of the defendant’s conduct (on what actually happens), rather than on its subjective character (on the intentions, beliefs, and practical attitudes which it displays).”^48^ Though inchoate offenses do not wear outcomes on their definitional sleeves, the *actus reus* requirement means that they do generate outcomes of some kind.

These outcomes can be specified in either of the two following ways. One could first stress that inchoate offenses typically result in psychological harms on the part of victims, like apprehension or fear. This specification strategy might seem extensionally imperfect, because some victims never become aware of the offenses that tentatively target them. However, with victims having the right to be notified of the arrest and, more commonly, the trial of the defendant, the probability of psychological harm caused by inchoate offenses remains prevalent.^49^

The second specificatory strategy overcomes these extensional limits. It emphasizes that the outcomes needed for criminalizing inchoate offenses are directly harmful to the victim but not actually experienced by her. The argument of direct non-experiential harms has been most notably defended by David Hershenov.^50^ Hershenov argues that a person can be harmed by an action even if she does not actually experience its harm. Hershenov gives the example of someone who never discovers the infidelity or suffering of a loved one.^51^ Another example is the case of a person whose bank account is, unbeknownst to him, constantly pilfered by someone with secret access to it. Yet another one is the kidnapping of a child who does not realize she is being kidnapped. Someone who acknowledges that these latter two examples can be properly interpreted as cases of harm will also need to accept the idea of non-experiential harms.

Note that the distinction between experiential and non-experiential harms might also offer a suitable basis for justifying our commonly held intuitions that inchoate offenses should be punished less than consummated ones.^52^ The principled consideration for this differential treatment is that experiential harms are typically more serious than non-experiential ones.

The aim of this sub-section was to show that there are plausible reasons for thinking that corrective justice can account for inchoate offenses. A more systematic defense of these reasons is, of course, needed. But, given the prefatory purpose of this article, a simple charting of these reasons should be sufficient for making a *prima facie* case for corrective justice as a principle of criminal law.

## Corrective Criminal Law: Two Objections

This last section will briefly try to dispel two more general worries one might raise about corrective justice as a principle of criminalization. The first worry is that corrective justice requires that we construe criminal law as private law. The thought runs as follows: because corrective justice is about the interests of private people, it will be unable to explain the public nature of criminal procedure or vindicate the claim that criminal law protects state interests. This claim is unwarranted in both of its claims. First, one cannot merely posit that criminal law is meant to protect state interests without further argument. Moreover, arguing that criminal law is essentially public might prove pernicious. This is because it would ground criminal law in a principle whose vagueness could (and sometimes does) err toward overcriminalization.^53^

As Dubber showed, the project of anchoring criminal law in the state’s public interests has a problematic pedigree. It goes back to the thesis that the function of the criminal law is to guard the sovereign’s peace *qua* person distinct from society as a whole. Thus construed, “an account of crime as an offence against the public peace is not an account of criminal law: it is an account of criminal police.”^54^ Today, some versions of the publicness thesis rest on a confusion. They amalgamate the assertion that, through the institutions of the state, members of the public “take an interest in crime” with a view of “crime directly violating the public’s interest.”^55^ Of these two assertions, the latter is false, because most crimes are neither committed in public nor serious enough to affect the public’s welfare considered as a whole. The claim about the public nature of criminal law cannot be *substantively* true.

The publicness claim is nonetheless procedurally true. This is to say that the state holds legitimate control over the application of criminal procedure. Unlike civil law, the victim is not a full party in the criminal law process. But this is not an institutional fact that is incompatible with the principle of corrective justice. Though the substance of criminal law should be about violations committed by an offender against a specific victim, the public nature of its procedure can be instrumentally justified. The state is, in virtue of its institutional resources and presumed impartiality, better placed to enforce justice than any individual victim can be. There is no straightforward inference that takes us from the thesis that, because crime is a violation of the victim’s interest, to the claim that the victim should have full control of the criminal justice process. Even in the case of civil law, there are procedural constraints grounded in the values of justice or efficiency with which the victim must comply.

Moreover, recent changes in the structure of criminal procedure express an increasing concern for victims’ interests. Though victims have been long excluded from the criminal justice process, they now enjoy a significant amount of procedural rights and safeguards. For example, victims are entitled to be informed about defendants’ arrests and arraignments, their appeals, releases, or escapes. Furthermore, they have a right to restitution from offenders, and are sometimes given a say in their probation or parole hearings.^56^ Given its focus on the relationship between offender and victim, corrective justice is particularly well placed for explaining this recent procedural dynamic in criminal law.

The second general objection about corrective justice is that it will erase the distinction between criminal and civil law. The thought is that, unlike civil law sanctions, criminal punishment is about harsh treatment, not about compensation. The contention here is that punishment should be defined in terms of the suffering that is intentionally imposed on the offender. I have criticized this thesis elsewhere, and will not develop my criticism here.^57^ Suffice it to say that building suffering into the *definiens* of legal punishment will tend to exclude forms of punishment—like compensation orders, reparation, or criminal restitution—that are primarily about correction, not about affliction.

More generally, this second objection seems to flow from the claim that correction is reducible to compensation. Since there are other defensible forms of correction—like reparation, reconciliation meetings, or apology—this claim is false. The emphasis on punishment as intentionally inflicted suffering, then, is surreptitiously grounded on the assumption that there is a privileged form of punishment—most likely, imprisonment—whose afflictive dimension should be present in other criminal sanctions as well. Given that imprisonment is not the most frequent criminal sanction, this assumption is descriptively false.^58^ Also, given the questionable moral record prisons have, the assumption is normatively shaky.

Though it is true that it will bring civil and criminal law normatively closer, corrective justice does not require that we treat torts or broken contracts as crimes. There is one important reason for keeping criminal law distinct from other areas of law. Given the nature and seriousness of the harms typically triggered by crime, the victim is not in a position to enforce and control criminal procedure. As Tadros argues, there is no deep principled difference between criminal law, on the one hand, and tort and contract law, on the other.^59^ But there are relevant differences between these areas of law, most of which have to do with the fact that punishment or the state’s more intrusive involvement are not needed to protect a person against the consequences of a broken contract or accidental injuries. Corrective justice can, for prudential reasons, leave a lot of these practical differences in place.

## Conclusion

The aim of this article was to show that corrective justice is an adequate principle for grounding criminal law. I focused on the MPC as the *justificandum* and on the equal private property claims between victim and offender as the *justificans*. I also argued that outcome-responsibility offers a promising platform for construing criminal liability. The structure of the article remains that of a prolegomenon and, as such, is exploratory rather than systematic.

There are cases—for instance, some regulatory offenses or victimless crimes—corrective justice cannot justify. Whether these limits are bullets worth biting or not, the specific limits of corrective justice point to a more general fact about any monist normative theory. The fact is that there are normative features of certain crimes or criminalizable actions any single principle of criminal law will be unable to account for. Even so, if a monist theory like the one provided by corrective justice can account for the main criminal cases, the fact that it cannot be extended to more liminal ones should not count as a flaw strong enough to reject the theory. The boundaries to its extension could be better interpreted as the justified limits of criminal law.

## Footnotes

1 For an analysis of the connection between corrective justice and private property, see A. Poama, “A Distinction with a Difference: Aristotle’s Division of Particular Justice and Its Practical Significance,” in E. Cohen de Lara, R. Brouwer (eds.), *Aristotle’s Practical Philosophy: On the Relationship between His Ethics and Politics* (Springer 2017).

2 D. Husak, *Overcriminalization: The Limits of the Criminal Law* (Oxford University Press, 2008) 10.

3 See also Mireille Delmas-Marty and Robert Badinter’s failed attempt to count the offenses in French criminal law, in M. Delmas-Marty, *Le flou du droit: du code pénal aux droits de l’homme* (Presses Universitaires de France, 2004). Similarly, the Committee on Justice quoted by the *Criminal Law: A Criminal Code for England and Wales (Law Com 177)*, vol. 1, para. 3.3, p. 12, stated that “it is now impossible to ascertain the content of the criminal law at any given time.”

4 I take Thom Brooks to be attempting something similar in T. Brooks, *Punishment* (Routledge, 2013), though I am less attracted than he is to the project of a “unified theory” of criminal law.

5 M. Dubber, *An Introduction to the Model Penal Code* (Oxford University Press 2015) 4.

6 This reason appears explicitly in the *Commentaries* to the UK Draft Criminal Code, 6.

7 For lack of space, I will refrain from a critique of retributive and preventive justice as grounds for criminal law, though such a critique could indirectly (though not conclusively) make my corrective justice justification more attractive.

8 R.E. Barnett, *The Structure of Liberty: Justice and the Rule of Law*. (Oxford University Press, 1988); C.F. Abel, F.H. Marsh, *Punishment and Restitution. A Restitutionary Approach to Crime and the Criminal* (Praeger, 1984).

9 N. Christie, “Conflicts as Property” (1977) 17(1) British Journal of Criminology 1–15.

10 R.A. Duff, “Virtue, Vice, and Criminal Liability: Do We Want an Aristotelian Criminal Law” (2002) 6(1) Buffalo Criminal Law Review 147–148.

11 There are some regulatory offenses for which corrective justice could account, especially cases where we are dealing with activities that require an increased degree of care.

12 For a discussion about the core-periphery approach to criminal law, see M.N. Berman, “Punishment and Justification” (2008) 118(2) Ethics 258–290.

13 This intuition is shared by most lay citizens. See G. Mitchell, P.E. Tetlock, “An Empirical Inquiry into the Relation of Corrective Justice to Distributive Justice” (2006) 3(3) Journal of Empirical Legal Studies 421–466.

14 E. Weinrib, *Corrective Justice* (Oxford University Press, 2012).

15 J. Coleman, *Risks and Wrongs* (Cambridge University Press, 2002).

16 Saying that Weinrib and Coleman focus only on interpersonal rights and interests is also insufficient, because, while it tells us something about their modality (i.e., about how those rights and interest are held), it does not say anything about their substance. Moreover, on an account where *all* rights and interests are ultimately interpersonal, a focus on their interpersonal dimension is not doing any specificatory work.

17
*Id.*, 333 ff.

18 Aristotle, *Nicomachean Ethics*, ed. by Jonathan Barnes, 2nd volume (Princeton University Press, 1984) 1130b34–35.

19
*Ibidem* 1130b37–38.

20
*Ibid* 1131a9.

21 For an analysis of the correspondence between the modes of responsibility in the MPC and Aristotle’s conception of voluntary action and blameworthiness, see L. Zaibert, “A Non-Aretaitc Return to Aristotle” (2011) 97(2) Archiv für Rechts-Und Sozialphilosophie 235–250.

22 T. Honoré, *Responsibility and Fault* (Hart Publishing, 1999); S. Perry, “Responsibility for Outcomes, Risk, and the Law of Torts” in G.J. Postema (ed.), *Philosophy and the Law of Torts* (Cambridge University Press, 2001). Toward the end of this chapter, Perry explicitly suggests that the idea of outcome-responsibility could be extended to criminal liability.

23 The connection between corrective justice and some form of personal responsibility is generally accepted as uncontroversial. For a critique of this connection that I will not engage with here, see A. Beever, “Corrective Justice and Personal Responsibility in Tort Law” (2008) 28(3) Oxford Journal of Legal Studies 475–500.

24 Perry, *supra* note 22, *idem* 80.

25 I will not examine how outcome-responsibility could ground strict liability in criminal law. Suffice it to say that, following the avoidability conception, outcome-responsibility could account for strict liability only for practices where extreme care is required. This seems to be consonant with the view the MPC adopts on strict liability, see MPC: *Commentaries*, 264.

26 This does not mean that corrective justice is based on an assessment of *other* dispositions (say, moderation or generosity) that might be part of a person’s character or of that person’s character considered as a whole. Corrective justice is tied only to the disposition that is relevant for guiding interactions between persons viewed as owners of private goods, i.e., to the disposition that makes a person morally sensitive to honoring what is “mine” and “yours” in interpersonal interactions. This specification of the relevant disposition is in line with Aristotle’s contention that ‘it makes no difference whether a good man has defrauded a bad man or a bad man a good one’, in Aristotle, *supra* note 18 1131b34–39.

27
*Idem* 1132a9–15.

28
*Idem* 1131b.

29 Aristotle considered it to be an injustice because he considered a person’s life to be at least in part the property of the city.

30 A. Ashworth, “Punishment and Compensation: Victims, Offenders, and the State” (1986) 6(1) Oxford Journal of Legal Studies 98.

31 This is particularly advantageous with recklessness being the default mode of responsibility both in English law and in the MPC. In *A Criminal Law for England and Wales*, vol. 1, *Report and Draft Criminal Law Bill* (1989), a person is typically considered “guilty of an offense” under a disjunctive condition that “he intentionally or recklessly causes” the material element of crime. This means that, in all these cases, recklessness is sufficient for establishing criminal liability.

32 V. Tadros, *Criminal Responsibility* (Oxford University Press, 2007) 84.

33 The adequate mode of sanctioning such violent offenses is obviously a central question that any theory of punishment needs to answer, but, despite its importance, it is not a problem that a theory of criminal law *needs* to answer fully, if we restrict that theory to the definition of substantive offenses and the nature of criminal liability.

34 These are different from offense types (that is, broad offense categories), and they refer to the numerically distinct offenses contained in the MPC. The difference is generated by the fact that one offense type (like homicide) might have several tokens.

35 Though notice that some others (like retaliation against a witness or informant) can be adequately justified on a corrective justice basis.

36 J. Mitchell, “Crimes of Misery and Theories of Punishment” (2012) 15(4) New Criminal Law Review 465–510.

37 Another hard case that I will not examine here is homicide.

38 D. Dripps, “Beyond Rape: An Essay on the Difference between the Presence of Force and the Absence of Consent” (1992) 92(7) Columbia Law Review 1780–1809. See also G.A. Cohen, *Self-Ownership, Freedom and Equality* (Cambridge University Press, 1995).

39 Dripps, *idem* 1797.

40
*Ibidem*.

41 J. Gardner, *Offences and Defences: Selected Essays in the Philosophy of Criminal Law* (Oxford University Press, 2008) 13.

42 For a critique of this type of conception, see especially D. Parfit, *Reasons and Persons* (Oxford University Press, 1984).

43 Gardner, *idem*, 3–5.

44 J.M. Bernstein, *Torture and Dignity: An Essay on Moral Injury* (University of Chicago Press, 2015) 127–128.

45 D. Dripps, “More on Distinguishing Sex, Sexual Expropriation, and Sexual Assault: A Reply to Professor West” (1993) 93(6) Columbia Law Review 1469–1470.

46 For an analysis about how granting rights has value-enhancing effects, see A. Harel, *Why Law Matters* (Oxford University Press, 2014).

47 For an interpretation of corrective justice as protecting one’s interest in one’s (good) name, see G. Scarre, “Corrective Justice and Reputation” (2006) 3(3) Journal of Moral Philosophy 305–319.

48 R.A. Duff, *Intention, Agency & Criminal Liability: Philosophy of Action and the Criminal Law* (Blackwell Publishing, 1990).

49 On victims’ rights, see P.M. Tobolowsky, *Crime Victim Rights and Remedies* (Carolina Academic Press, 2001). One might worry that recognizing psychological harm opens the door to overcriminalization. This is not necessarily the case if we set the standard for the kinds of psychological harm that would constitute corrective wrongs high enough and if, in addition, we are able to design a test that establishes the physical injury effects of psychological harm. For a more detailed discussion on the latter point, see G.B. Melton et al., *Psychological Evaluations for the Courts. A Handbook for Mental Health Professionals and Lawyers*, 3rd ed. (Guilford Press, 2007) 413 ff.

50 D.B. Hershenov, “Restitution and Revenge” (1999) 96(2) Journal of Philosophy 79–94.

51
*Idem*, 88.

52 P.H. Robinson, J.M. Darley, *Justice, Liability and Blame: Community Views and the Criminal Law* (Westview Press, 1996).

53 M. Dubber, “Criminal Law between Public and Private Law” in R.A. Duff et al. (eds.), *The Boundaries of the Criminal Law* (Oxford University Press, 2010) 208.

54
*Ibidem*.

55
*Idem* 210–211.

56 Tobolowsky, *supra* note 49.

57 See *supra* note 1.

58
*Idem.*

59 V. Tadros, *The Ends of Harm. The Moral Foundations of Criminal Law* (Oxford University Press, 2011) 279.

